# ExtraCECI: a community-based person-centred-enhanced care intervention to improve the quality of life and person-centred outcomes for people living with HIV/AIDS in Ghana—protocol for cluster randomised controlled trial

**DOI:** 10.1136/bmjopen-2025-102692

**Published:** 2025-05-23

**Authors:** Mary Abboah-Offei, Helen Elsey, Kennedy Bashan Nkhoma, Samson Abboah-Offei, Ada Keding, Justin Fenty, Antonina Yakimova, Catherine Hewitt, Gladys Dzansi, Vivian Efua Senoo-Dogbey, Stephen Ayisi Addo, James Akazili, Andrews Ayim, Richard Harding, The ExtraCECI Project Team

**Affiliations:** 1 School of Health and Social Care, Edinburgh Napier University, Edinburgh, Scotland, UK; 2 Department of Health Sciences, Hull and York Medical School York, University of York, York, UK; 3 Florence Nightingale Faculty of Nursing Midwifery and Palliative Care, King’s College London, London, UK; 4 Health Sciences, University of York Faculty of Sciences, York, UK; 5 School of Nursing and Midwifery, Adult Health, University of Ghana College of Health Sciences, Accra, Greater Accra Region, Ghana; 6 Public Health Nursing, University of Ghana, Accra, Greater Accra, Ghana; 7 National HIV/AIDS Control Programme, Ghana Health Service, Accra, Greater Accra Region, Ghana; 8 School of Public Health, C K Tedam University of Technology and Applied Sciences, Navrongo, Upper East Region, Ghana; 9 Ghana College of Physicians and Surgeons, Accra, Greater Accra, Ghana

**Keywords:** Clinical Trial, Health Services, HIV & AIDS, Public health, Patient Reported Outcome Measures, Person-Centered Care

## Abstract

**Introduction:**

People living with HIV/AIDS (PLWHA) have complex physical, psychological, social and spiritual needs following diagnosis and poorer health-related quality of life than the general population. Holistic assessment and care delivery incorporating person-centred principles is required to address these needs. This protocol describes a cluster randomised controlled trial (cRCT) and process evaluation to evaluate the effectiveness of the extra community-based enhanced care intervention (ExtraCECI) to improve the quality of life and person-centred outcomes for PLWHA in Ghana.

**Methods and analysis:**

This cRCT will randomly assign 26 recruited HIV clinics using 1:1 allocation to either ExtraCECI intervention or standard HIV care, with each clinic recruiting an average of 25 participants, that is, 650 in total. Eligible participants are adult PLWHA aged at least 18 years and in HIV care for at least 6 months, with cognitive ability to consent as guided by the Mental Capacity Act, clinically well to participate, attending an outpatient clinic. Healthcare professionals (HCP) at clinics randomised to the ExtraCECI intervention arm will receive training on person-centred care and holistic assessment of PLWHA in the domains of physical, psychological, social and spiritual well-being. PLWHA will be empowered to contribute to their care decisions including HCP using telehealth for ExtraCECI delivery with ongoing mentorship, while participants in the Standard HIV Care arm continue with usual care. The primary outcome is quality of life measured at the individual level using Medical Outcomes Study-HIV (MOS-HIV). The primary analysis will compare MOS-HIV total scores between groups using repeated measure linear mixed model and adjusting for important baseline characteristics (including stratification factors) and random effect of clinic. The incremental cost-effectiveness ratio will be used to estimate the cost-effectiveness of the ExtraCECI intervention, and a process evaluation will be conducted.

**Ethics and dissemination:**

This protocol was approved by Edinburgh Napier University School of Health and Social Care Research Integrity Committee (REF: SHSC3681836) and the Ghana Health Service Ethics Review Committee (GHS-ERC:010/07/24). Results from this study whether positive or negative will be presented to participating sites, communities, at scientific conferences and published in peer-reviewed journals.

**Trial registration number:**

ISRCTN77405303.

**STRENGTHS AND LIMITATIONS OF THIS STUDY**

- The study will use a cluster randomised controlled design to evaluate the effectiveness of the extra community-based enhanced care intervention (ExtraCECI), this design minimises the risk of confounding factors influencing the results.
- The training component of the ExtraCECI intervention allows for the training of health professionals at the clinic level, building their capacity to continue to deliver person-centred care beyond study end.
- The 26 clusters being included in this study are in the Greater Accra Region of Ghana, thus extrapolating findings to other regions remains to be explored, however, with increased urbanisation; findings are likely to be relevant to other areas of Ghana.
- Due to the nature of the intervention, it is not possible to blind participants or researchers as this is a behavioural intervention and outcomes are self-reported.

## Introduction

 HIV/AIDS remains a condition of public health concern with approximately 39.9 million people living with HIV and about 30.7 million receiving antiretroviral therapy (ART) globally.^1^ Sub-Saharan Africa carries more than two-thirds of the global HIV burden. There are an estimated 330 000 people living with HIV in Ghana.^2^ People with HIV have complex physical, psychological, social and spiritual needs following diagnosis^3^ and poorer health-related quality of life (QoL) than the general population.^4^ Self-reported physical and psychological symptoms are associated with poorer ART adherence,^5^ sexual risk taking,^6^ viral rebound^7^ and poorer self-rating of health.^8^ In contrast, good psychosocial care and communication with HIV care providers are associated with improvements in clinical outcomes, adherence and retention in care.^9 10^ The Joint United Nations Programme on HIV and AIDS (UNAIDS) advocates for countries to adopt community-based approaches and to keep people living with HIV/AIDS (PLWHA) ‘healthy and alive through the delivery of person-centred and holistic care’.^11^ Task shifting and decentralising HIV services to community settings in low and middle-income countries (LMICs) have proven cost-effective,^12^ resulting in decreased HIV-related mortality, virologic failure and other adverse health outcomes.^13^,^16^

UNAIDS proposed person-centred care (PCC) as a fast-track action for its ‘95-95-95’ global strategy to improve rates of diagnosis, treatment adherence and viral suppression for PLWHA by 2030.^17^ With the 95-95-95 targets, Ghana currently stands at around ‘72-87-68’ for diagnosis, treatment and viral suppression, respectively.^18^ Respondents to this strategy, including HIV community representatives, advocacy groups and experts in the field, have also advocated for a fourth priority focusing on improved QoL.^19^ Greatest attention has been paid to viral suppression, at the expense of broader psychological, social and spiritual concerns that persist despite treatment advances.^6 20^ Care that addresses the multidimensional concerns of people with HIV requires a person-centred approach, a core principle of quality healthcare.^21 22^ Mezzich defined PCC as care ‘dedicated to the promotion of health as a state of physical, mental, social and spiritual well-being as well as to the reduction of disease and founded on mutual respect for the dignity and responsibility of each individual person’.^23^ Thus, PCC puts the individual at the centre of their care, helps individuals to access the care they need, when they need it, by involving them in their own care decisions.^24^ Such approaches have been found to improve patient experience, care quality and health outcomes.^25^ However, PCC is an approach that evolved in high-income settings, and there are limited data available to model contextually and culturally appropriate PCC in LMIC.^26^,^28^ A clinical trial in Kenya reported that the use of person-centred assessment and care delivered by trained healthcare professionals (HCP) to people with HIV on treatment had a positive effect on self-reported mental health-related QoL and psychosocial well-being.^29^

In order to generate evidence for feasible practice of PCC in Ghana, we followed the Medical Research Council (MRC) guidelines for developing and evaluating complex interventions^30 31^ to first identify the evidence of person-centred models of HIV care in community settings.^32^ Then using Mezzich’s definition as our theory of PCC,^23^ we explored what constitutes PCC for PLWHA and HCP as well as what outcomes matter to them.^33^ Findings from these studies informed the development of a community-based enhanced care intervention (CECI) to improve person-centred outcomes for PLWHA, which was tested in a feasibility cluster randomised controlled trial (cRCT).^34^ CECI feasibility testing achieved recruitment and retention rates with post-trial interviews reported good acceptability among PLWHA as well as highlighted important areas of refinement to CECI.^34^

### Aim and objectives

To conduct a cRCT to evaluate the effectiveness of a person-centred intervention for people with HIV (extra community-based enhanced care intervention; ExtraCECI) compared with standard HIV care in improving QoL and person-centred outcomes for PLWHA in Ghana.

### Objectives

1. To establish a patient and public involvement (PPI) person-centred network of PLWHA support groups and stakeholders to inform research procedures and dissemination throughout the project implementation.
2. To refine the CECI by adding an extra component to become ‘ExtraCECI’ through a theory of change workshop.
3. To evaluate the effectiveness of ExtraCECI to improve QoL and person-centred outcomes for PLWHA compared with standard HIV care.
4. To assess the cost-effectiveness of ExtraCECI compared with standard HIV care and describe the implications for Ghana Health Services’ resource management.
5. To conduct a process evaluation using quantitative and qualitative data sources to understand facilitators and barriers to implementation and identify what worked, for whom, why and in what circumstances.

## Methods and analysis

### Setting

This study will be conducted in community HIV clinics within the Greater Accra Region (GAR) of Ghana. The region has both urban and rural communities with a mixture of different socioeconomic, cultural and educational backgrounds. Of the 346,120 PLWHA in Ghana, about 76,730 PLWHA are in the GAR,^35^ with approximately 94 ART clinics and 506 HIV testing facilities. Eligible clinics provide similar HIV care services including provision of ART, care for key populations (men who have sex with men (MSM), sex workers and drug users), ART adherence, counselling, psychosocial and spiritual care.

### Establishment of patient and public involvement

We will work with community health workers, clinic staff and Models of Hope (PLWHA peer supporters) within the GAR of Ghana to identify community leaders of PLWHA advocacy and support groups. The Models of Hope are not available at some clinics however this project will recruit 20 PLWHA peer supporters to cover multiple clinics during the project implementation. Our public and PLWHA group will be drawn from these advocacy and support groups and will be engaged throughout the research processes from study inception, CECI refinement with associated study documents, project delivery and the interpretation of trial findings. The public and PLWHA group will also serve as an advisory group and will be provided with introductory training on research and the principles of PCC to enable them to effectively discharge their role.

#### Refining CECI to ‘ExtraCECI’

The CECI components include: (1) a training programme on person-centred communication, (2) holistic assessment of PLWHA’s symptoms and concerns in the domains of physical, psychological, social, and spiritual well-being using a structured tool, (3) a care plan to capture holistic needs to facilitate collaborative care planning and delivery, (4) regular support/mentorship for HCP with fidelity monitoring.

‘Extra’ components of: (1) empowering PLWHA on how to engage, participate and contribute to their care decisions; and (2) working with HCP and Models of Hope to use telehealth (text messaging for information sharing and voice calls for follow-up, assessment and enabling feedback in the communication process) for care delivery is being added to CECI as ‘extra’ components. These extra components of CECI will rely on collaborative discussion of PLWHA’s symptoms and concerns with the HCP to inform holistic care planning and delivery. Consideration has been given to these additional components, as both HCP and PLWHA have identified the need for PLWHA to be empowered to be able on to engage, participate in care consultations and contribute to care decisions.^34^ Working with HCP and Models of Hope to use telehealth to deliver CECI gives PLWHA more options to access care through telecommunication.

The process of refinement will include:

1. CECI Theory of Change (ToC) workshop with stakeholders including our public and PLWHA group members, where CECI will be presented with the mechanisms of action identified during the feasibility cRCT.
2. The areas of refinement as identified as empowering PLWHA on how to engage, participate and contribute to their care decisions will be discussed with stakeholders for their views and input.
3. Additionally, the ToC workshop will explore how to work with HCP and Models of Hope to use mobile phones^36^ to deliver CECI, since mobile phones (eHealth) have shown to be effective in delivering interventions to PLWHA in sub-Saharan Africa.^37^
4. Share samples of the holistic assessment and care planning tools for CECI with stakeholders for review and input.

### ExtraCECI

Once the ‘Extra’ components of ‘empowering PLWHA to engage, participate and contribute to their care consultations and decisions’ and the use of telehealth have been added to CECI, the refined CECI will then be referred to as ExtraCECI. These extra components of empowering PLWHA will be incorporated at the clinic level pre-consultation meetings, where groups of PLWHA scheduled for their appointments usually have initial general discussions with HCP prior to having individual consultations. ExtraCECI aims to improve confidence levels of PLWHA to contribute meaningfully to their care decisions.

### Study design

**Figure 1:**
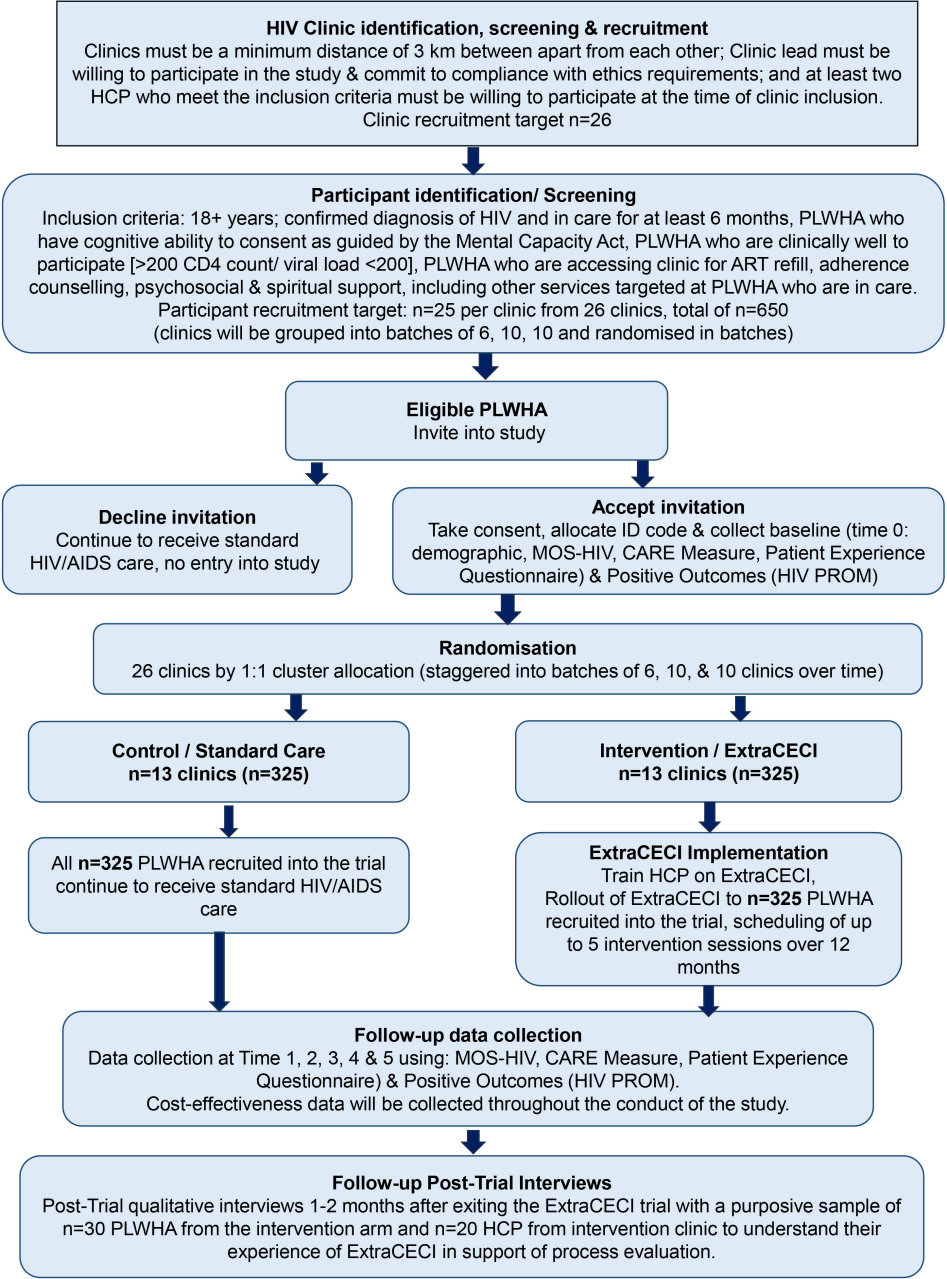
ExtraCECI study flowchart.

We will conduct a cRCT to evaluate the effectiveness of ExtraCECI in HIV clinics within Ghana. A parallel group design with 1:1 allocation ratio will be utilised including an economic evaluation and an embedded qualitative process evaluation informed by the ‘Evaluation stage’ of MRC guidelines for developing and evaluating complex interventions.^30 31^ Recruitment and baseline (T0) data collection at each cluster will be conducted before randomisation and the start of any intervention delivery. The first care appointment following randomisation will be treated as time point T1, followed by 3-monthly follow-ups at 3-, 6-, 9- and 12 months recorded at T2, T3, T4 and T5. The cRCT will be reported according to the CONSORT statement^38 39^ and details of study recruitment and participation presented in a CONSORT flow diagram as in figure 1.

#### Inclusion and exclusion criteria

The study inclusion criteria for clinics, HCP and PLWHA are presented in table 1.

**Table 1:** ExtraCECI study inclusion and exclusion criteria

Inclusion criteria	Exclusion criteria
HIV clinics Clinics with a minimum travel distance of 3 km apart to minimise treatment ‘contamination’^56^ between the intervention and control arms. The clinic lead must be willing to participate, be randomised and commit to compliance with ethics and study requirements. At least two HCP per clinic who meet the inclusion criteria (below) must be willing to participate at the time of clinic inclusion.	Clinics that are less than 3 km travel distance apart with potential for treatment contamination. Clinics not willing to participate in the study and/or not willing to comply with ethics and study requirements. Clinics with less than two HCP who meet the inclusion criteria at the time of clinic inclusion.
HCP HCP who regularly provide hands on care for PLWHA. HCP providing care for PLWHA in eligible clinics for at least 6 months. Willing to attend intervention training, mentorship and support sessions.	HCP not providing regular hands-on care to PLWHA. HCP providing care for PLWHA in eligible clinics for less than 6 months. Not willing to attend intervention training, mentorship and support sessions.
PLWHA Adults PLWHA from age 18 years. HIV positive diagnosis and in care for at least 6 months, to ensure that PLWHA has the experience of care to reflect on. PLWHA who have cognitive ability to consent as guided by the Mental Capacity Act. PLWHA who are clinically well to participate (having>200 cells/mm^3^ CD4 count/ viral load<200 without complications). PLWHA attending clinic for ART refill, adherence counselling, psychosocial and spiritual support, including other services targeted at PLWHA who are in care.	PLWHA under age 18 years. Diagnosed with HIV and in care for less than 6 months. PLWHA not having cognitive ability to consent. Having<200 cells/mm^3^ CD4 count or viral load>200, severe complications/ co-morbidities including cardiovascular diseases, malignancies, pneumonia and requiring specialised treatment at secondary/tertiary health facility). Persons attending clinic for pre-counselling & testing services including prevention of mother-to-child transmission.

### Participant screening consent and recruitment

HIV clinics within the Greater Accra Region of Ghana will be profiled based on their location, services provided and total number of PLWHA registered per clinic. Eligible clinics will be invited into the study with letters including the study information and consent forms. HCP within recruited clinics will initially screen potential participants and refer eligible participants to the researchers for further screening and study information discussion. Up to one week will be allowed for addressing questions from potential participants, and eligible participants who agree to take part in the research study will be asked to give informed consent by signing or thumb printing a consent form. This consent process will continue until the target number for each cluster (see the Site selection and randomisation section) is recruited. For those who provide informed consent to participate in the trial, their baseline data will be collected immediately or otherwise scheduled within one week of consenting for baseline data collection.

### Sample size

As there is no widely established minimal clinical important difference for the Medical Outcomes Study-HIV (MOS-HIV) measure, we followed best practice guidelines^40^ using a standardised effect size approach. We estimated that an average cluster would be 20, but there would be variation in this estimate, which we incorporated in the sample size (coefficient of variation).^41^ No clinic level intracluster correlation coefficient (ICC) is available, and the feasibility trial conducted was too small to reliably estimate the ICC, hence we chose a value of 0.04.^42^ Therefore, for 90% power to detect a minimum effect size of 0.4 in the MOS-HIV, a total of 650 PLWHA would need to be randomised from 26 clinics, assuming an ICC of 0.04, average cluster size of 25 with 20% loss to follow-up.

### Site selection and randomisation

The 26 HIV clinics recruited will be randomly allocated to either standard HIV care or ExtraCECI after baseline data collection and before intervention training for HCP using a restricted randomisation frame in three separate batches (6, 10 and 10 sites) in order to stagger recruitment and follow-up. A statistician from York Trials Unit will generate a randomisation frame consisting of a list of all possible combinations of allocating 26 sites to 2 arms using bespoke code in Stata V.18. The list of allowable combinations will be reduced by excluding combinations that do not correspond to a 1:1 allocation of sites within any batch. One combination will then be selected at random from the final restricted list. Allocation will only be communicated to sites once recruitment for the relevant batch is complete. See figure 1 for study flowchart.

### Blinding

Given the nature of the intervention, it is not possible to blind participants, HCP or researchers accessing outcomes to the intervention, as outcomes are self-reported.

### Standard HIV care

Clinics randomised to the control arm will continue to provide standard HIV care as the comparator throughout the cRCT. The standard HIV care consists of PLWHA attending their clinic appointments for either repeat prescription or for referrals to ART adherence support, nutrition support, CD4 count/viral load testing and for specific problem(s) as described by appointment note. These clinic appointments will be made to align with the 3 monthly follow-ups in the ExtraCECI arm.

### Intervention (ExtraCECI)

The ExtraCECI as described above will be targeted at both cluster and individual participant level. The difference between standard HIV care and ExtraCECI is that, while standard HIV care focuses mainly on the management of physical symptoms and ART services, ExtraCECI focuses on the holistic assessment and management of the physical, psychological, social and spiritual well-being of PLWHA in addition to ART services.

#### At cluster level

HCP will receive five sessions of intervention training delivered by MA-O supported by designated study team members at agreed times using a hybrid approach (virtual and face-to-face sessions) with PowerPoint presentations and role plays using sample healthcare scenarios for practice sessions. HCP will also receive regular mentorship support after each intervention delivery including monitoring of their stress and coping levels using Occupational Stress Scale. On completion of ExtraCECI training, there will be an embedding period where HCP will continue to practice the intervention at site for approximately 4 weeks, before intervention sessions will be delivered to all recruited PLWHA based on their respective appointments.

#### At the individual participant level

At their first intervention appointment, the researcher/HCP will provide the participant with a patient education leaflet focused on ways to support/empower them for their clinical consultation, for example, how to have a meaningful conversation about their symptoms and concerns with the HCP for collaborative care planning. HCP will discuss symptoms and concerns, collaboratively plan care based on identified needs using a structured client holistic assessment form and care plans to set short/long-term goals, prioritising needs and care goals. Optimal management approach will be used and action plans individualised to achieve small realistic goals by next appointment. Postcare assessments will happen at the clinic where PLWHA will have a discussion with the researcher for care outcome assessment.

### Primary and secondary outcomes and measures

All outcomes will be assessed by designated research assistants at baseline (T0), and after each scheduled appointment: the first postrandomisation appointment (T1) and at 3, 6, 9 and 12 months thereafter (T2, T3, T4 and T5, respectively). The primary outcome is self-reported QoL measured at the individual level using MOS-HIV^43^ total score at 12-month time point. The 35-items address the domains of role function, pain, physical functioning, cognitive functioning, social functioning, general health perception, mental health, distress and vitality. The secondary outcomes will include the MOS-HIV domain scores and total score at other timepoints and positive outcomes HIV PROM,^24 44^ a 23-item patient-centred PROM that reflects the range of outcomes relevant for PLWHA to drive and evaluate care. Picker Patient Experience^45^ is an 18-item self-reported measure, which measures patient experience along the domains of communication; emotions; short-term outcomes; barriers and relations with HCP. Consultation and Relational Empathy^46^ measure, a 10-item person-centred process questionnaire that measures the amount of empathy that a patient feels they have received during a consultation. Table 2 presents the schedule for data collection for outcome measures.

**Table 2:** Schedule for data collection

Data	Time point collected for both intervention and control							2 months post-trial
	Baseline	Time 1	Time 2	Time 3	Time 4	Time 5	Trial ends	
Eligibility data	✓							
Consent and contact details	✓							
Demographic and HIV history	✓							
Medical outcome study—HIV (MOS-HIV) for QoL	✓	✓	✓	✓	✓	✓		
Positive outcomes: HIV PROM—person-centredness	✓	✓	✓	✓	✓	✓		
CARE measure—person-centred process	✓	✓	✓	✓	✓	✓		
Picker Patient Experience Questionnaire (PPE-15)	✓	✓	✓	✓	✓	✓		
ExtraCECI related cost data	✓	✓	✓	✓	✓	✓		
Qualitative interviews								✓
Adverse event reporting	Ongoing							
Process evaluation	Ongoing							
Economic evaluation	Ongoing							

ExtraCECI -, Extra Community-based Enhanced Care Intervention.

### Participant compliance, loss to follow-up and withdrawal

No participant will be withdrawn from the trial based solely on non-compliance to the intervention. Participants may withdraw from the study at any time without influencing their future care.

A withdrawal form will be completed when a PLWHA does not wish to continue participating in the intervention and or trial-related activities including follow-up assessments. The withdrawal forms will be completed by research assistants/HCP, and all withdrawal data will be uploaded to Qualtrics, hosted by the York Trials Unit. We will contact HCP to request new contact details of any participant for whom we have lost contact.

### Adverse events

The ExtraCECI study is a behavioural intervention trial, with no medicinal product being used and thus, no medical or physical health adverse events (AEs) are expected. However, there is a low risk that conversations around psychosocial well-being could trigger distressing emotions and thoughts, which could trigger emotional breakdown. As such, researchers will be vigilant in looking out for the impact of the study on the well-being and psychological safety of study participants. For the purposes of the ExtraCECI intervention trial, AEs are defined as any untoward medical occurrence (ie, any unfavourable and unintended sign, symptom or disease), experienced by a trial participant, which is temporally associated with trial treatment (intervention or control). AEs, which might be expected, include potential general discomfort associated with responding to questionnaires, social, economic and psychological impacts. AEs which would not require reporting include medical conditions such as stroke, heart attack, accidents, infections and all other conditions requiring emergency treatment or admission for general medical services.

### Contamination

Using clusters as a unit of randomisation and maintaining a 3 km travel distance between clinics should minimise the risk of contamination. However, there is a theoretical possibility of some contamination if PLWHA in the ExtraCECI arm was to meet with PLWHA in the standard HIV care arm and then divulge information about intervention components. To minimise the possibility of any form of contamination, participants in both clusters will be asked if they visited any health facility since the last appointment. We will request those in the ExtraCECI (intervention group) to refrain from sharing study-related information and materials during the study. We will also ask those in the standard HIV care arm if they had any contact with any participant from the ExtraCECI arm.

### Data management

Data management will be in accordance with the General Data Protection Regulation (GDPR)^47^ and Ghana Data Protection Act 2012.^48^ Each site and participant will be assigned a unique trial identification number at the start of data collection. A record sheet linking patient details and identification number for participants including consent forms will be kept at each site in a securely locked filling cabinet, separate from datasheets.

Quantitative outcome data will be collected using paper-based questionnaire booklets for each participant, and the hard copies of the assessments will be stored securely at clinic sites or where possible inside a locked cabinet at a secured office at the University of Ghana (UoG). Each participant CRF booklet data will be entered into Qualtrics (online version), hosted by the UK Clinical Trials Unit. The in-country data manager will audit the data entry process. The resulting database will only be accessible by the team at the Clinical Trials Unit, who will query the data entered by sites as well as checking for completeness and consistency. Data sharing agreements will be put in place for the transfer, storage, restricted access and disposal of personal information in accordance with GDPR,^47^ Ghana Data Protection Act 2012,^48^ Clinical Trials Unit Standard Operating Procedures (SOP) and Good Clinical Practice (GCP) regulations.

### Statistical and effectiveness analysis

Analyses will be undertaken in Stata V.18 (or later) based on two-sided 5% significance levels under the principles of intention-to-treat, as detailed in a statistical analysis plan. Baseline data will be summarised descriptively by treatment group using means and SD or median and IQR for continuous variables and counts and percentages for categorical variables. Participants' MOS-HIV scores will be analysed using a repeated measures linear mixed effects model. The model will include MOS-HIV scores as outcome, with fixed effect of treatment by time interaction, adjusting for important baseline variables (including stratification factors) and random effect of clinic. Time points will be nested within patients and modelled using a covariance structure. Different structures will be considered and the simplest structure with the best fit (assessed using Bayesian Information Criterion) will be used. Model assumptions of normality of standardised residuals will be assessed (using Q-Q plots) in addition to homoscedasticity (using a scatter plot of standardised residuals against fitted values). If the assumptions are not met, relevant transformations of MOS-HIV scores and non-parametric tests will be considered. The treatment effect at the time points will be extracted in the form of an adjusted mean difference, 95% CI and p value (with the primary being at 12 months). Analysis of the primary outcome will be checked by a second statistician in accordance with clinical trials unit SOPs (Primary Analysis Sign Off Form) before results are circulated to wider members of the trial team. Continuous secondary outcomes will be similarly analysed using a similar model to the primary analysis.

### Cost-effectiveness evaluation

We will adapt an existing costing measure for the economic evaluation of ExtraCECI from HCP perspective and reported by the Consolidated Health Economic Evaluation Reporting Standards (CHEERS) 2022 checklist.^49^ The effectiveness will be determined by self-reported QoL in the MOS-HIV measurements (descriptions) and valuations (utilities). For QoL measurements, the overall mean scores and scores for each domain of the MOS-HIV will be calculated. Analysis will be carried out to determine the difference between the overall mean scores of each in the subgroups of treatment (intervention and control).

Cost data will be collected from all the HIV study clinics (intervention and control) on activities associated with HIV management as well as the intervention-specific activities. This will require taking inventory of all resources in the facilities and then allocate these costs to HIV management cost centre, using CHEERS 2022 checklist,^49^ to guide the data collection and analysis. A 1-year time horizon will be applied for both cost and effectiveness. We will use standard microcosting techniques, incorporating a full costing approach, with both recurrent costs, overhead items and capital costs.

We will annualise capital costs at a discount rate of 3% per year, and according to the useful life of each item.^50^ The total economic costs of ExtraCECI will be estimated and then categorised into preintervention, intervention and indirect costs. The incremental cost-effectiveness ratio, for the estimation of the cost-effectiveness, will be expressed as the ratio of the difference in cost to the difference in effect (QoL) between ExtraCECI versus standard HIV care. Furthermore, a Budget Impact Analysis will be conducted to gauge the impact of the intervention on governmental health expenditure.^51^

One-way sensitivity analysis will be conducted to determine whether changes in variables (discount rate and life expectancy of equipment) and PLWHA utilisation of ExtraCECI will change the economic costs or cost-effectiveness of the implementation. Accordingly, the discount rate will be varied from 3% to 10% as well as the life span for equipment from 3 years to 10 years. Also, PLWHA utilisation will be varied using 5% to 10% higher and lower to determine the unit cost variations.

### Process evaluation (qualitative)

**Figure 2:**
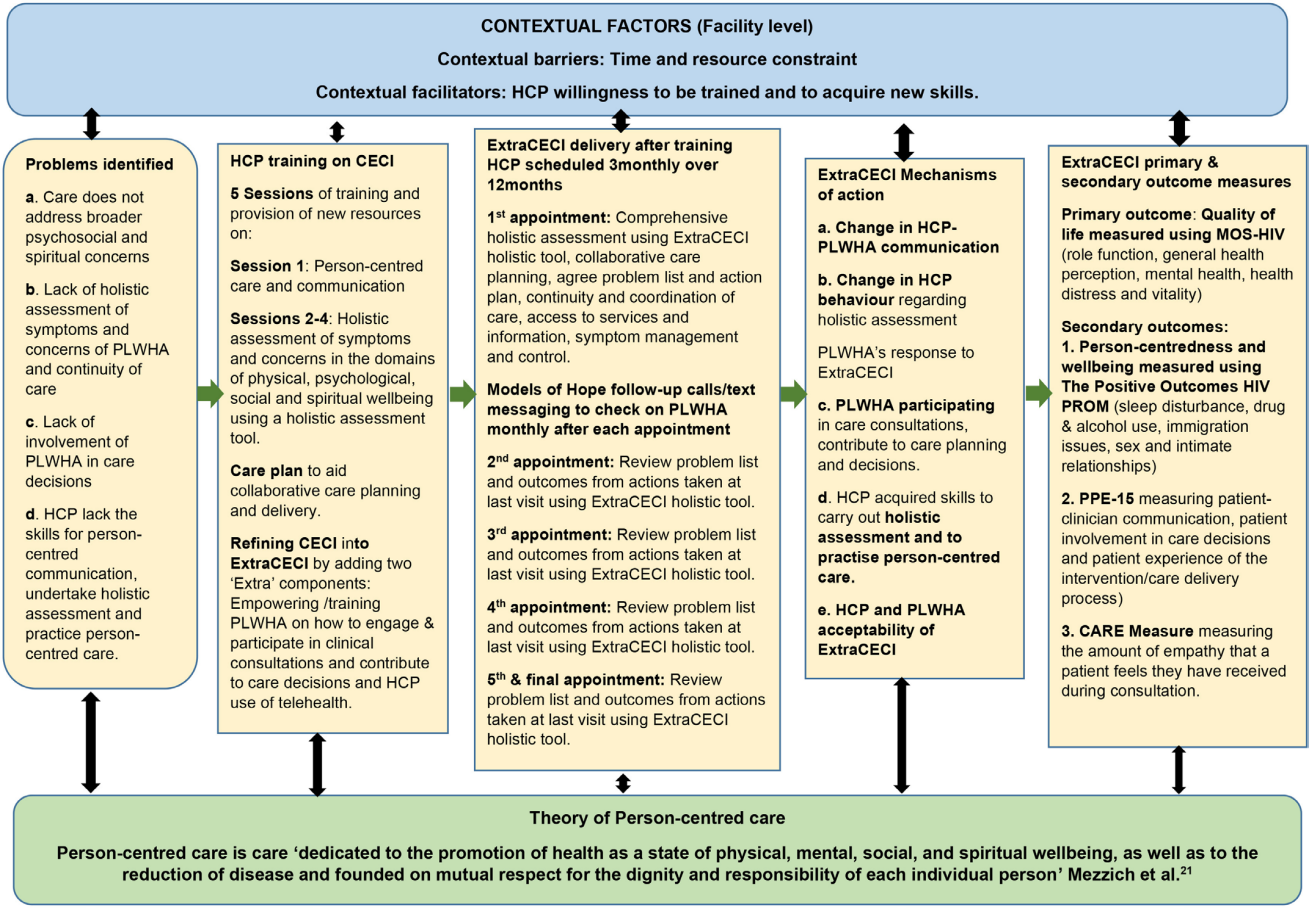
Logic model of ExtraCECI - Extra Community-based Enhanced Care Intervention.

A process evaluation will be conducted to understand the fidelity of the delivered ExtraCECI compared with our completed Template for Intervention Description and Replication checklist.^52^ The process evaluation will also clarify causal mechanisms and identify any facilitators or barriers to implementation as well as contextual factors linked to outcomes.^53^ The process evaluation will use data from post-trial qualitative interviews to explore the mechanisms of actions outlined in figure 2. Post-trial interviews will be conducted with PLWHA who received ExtraCECI sampled from at least five clinics (n=30) and HCP who delivered ExtraCECI and their managers (n=20), who will be purposely sampled from the main trial ensuring diverse characteristics, particularly gender, occupation and HIV management. These participants will be interviewed for 40–60 min following a semistructured guide. The interview data will be collected using digital encrypted audio recorders with notes taken as appropriate.

The recordings will be transferred onto a password-protected laptop for transcription, and the audio files subsequently deleted from the recorder. Interviews will be transcribed verbatim and translated into English where necessary. Anonymised transcripts will then be imported into NVivo (hosted by ENU) for thematic analysis. The stages of Clarke *et al*^54^ thematic analysis will be followed: familiarisation, generating codes, constructing themes, reviewing themes, and producing the findings. Transcripts will be analysed using deductive-inductive combination (hybrid approach) in thematic analysis proposed by Clarke *et al*^54^ to thoroughly understand ExtraCECI fidelity, implementation and impact. Data will be reported in line with the Consolidated Criteria for Reporting Qualitative Research checklist.^55^ The data obtained will complement the trial data to help identify the contexts and mechanisms that lead to positive outcomes in the trial.

### Joint trial steering committee and data monitoring committee

Due to the low risk profile of the ExtraCECI intervention, a combined steering and data monitoring committee (DMC) will be sought. Therefore, a trial steering committee (TSC) will be convened to provide overall supervision of the trial, ensuring its conduct is in accordance with the protocol and relevant regulations for trial safety. Within the TSC, a smaller DMC will monitor adverse and serious AEs related and unrelated to the trial. The committee will consist of an independent chair and at least two other independent members including a statistician and two context and subject expert, and one PPI, along with the PI, the trial manager and representative from the clinical trials unit. Other study collaborators may also attend the meeting at the discretion of the Chair. The TSC/DMC will meet two times a year to discuss progress of the trial, or more often as appropriate. The role of this committee will also include the review of all serious AEs, and the AEs which are thought to be treatment-related and unexpected.

### Trial management

The day-to-day management of the trial will be undertaken by the trial management group (TMG), which will be chaired by the principal investigator (PI) and will include the trial coordinator, local PIs, coinvestigators and trial statisticians. Monitoring the conduct of the trial and related activities (including the health economic evaluation and process evaluation) will be provided by the TSC. The TSC will include three/four independent experts whose responsibilities will include advising the TMG on any trial management, data management/analysis, ethics and safety monitoring. The trial is being sponsored by Edinburgh Napier University (ENU). A representative of the Sponsor and funder will be invited to attend the TSC meetings.

## Ethics and dissemination

### Research ethics approval

The trial protocol was approved by the ENU School of Health and Social Care Research Integrity Committee (REF: SHSC3681836) and the Ghana Health Service Ethics Review Committee (GHS-ERC:010/07/24). The trial will be conducted according to the approved study protocol and following GCP guidelines and all study participants will sign the consent form (see online supplemental appendix 1) prior to participation. Any substantial deviations from the protocol will be sent to the regulatory authorities for prior review and approval. Appropriate protocol is in place to support any psychological distress experienced by participants including a distress protocol developed to guide researchers in the support process and possible onward referral for further management. The assessment of the trial AE will be conducted by the delegated clinical psychologist who becomes aware of the event and supported by HCP.

### Dissemination

The trial protocol, the trial results, intervention manual and cost-effectiveness results will be published in peer-reviewed open-access journals. Results will also be disseminated via study websites, policy briefs, blogs, social media and at scientific conferences.

### Capacity building

A capacity strengthening needs assessment of the study team with particular focus on development of early career researchers will be undertaken to inform a capacity strengthening plans and ensuring it is contextually appropriate and relevant. Furthermore, capacity building activities will focus on meeting the practice and research gap in PCC by working in collaboration with the Ghana College of Nurses and Midwives, the Nursing and Midwifery Council and other HCP registration bodies to adopt ExtraCECI training as a CPD course for all registered HCP. This will serve as the starting point for bridging the PCC skills gap among HCP. Also, there are plans to build capacity around the conduct of clinical trials by exploring existing resources towards the development of an SOPs for a potential centre for clinical trials at the UoG. The study will also explore and discuss the potential for a postgraduate degree programmes or modules in clinical trials and health statistics as a starting point. The study will use existing links to facilitate engagement with local and global policy-makers to enhance PCC for PLWHA.

### Trial sponsor

Edinburgh Napier University, Head of Research Environment and Services, Sighthill Campus Room 7.B.14, 9 Sighthill Court, Edinburgh, EH11 4BN, g.barkess@napier.ac.uk

## Supplementary material

10.1136/bmjopen-2025-102692 online supplemental file 1
