# Supplementary material for: ExtraCECI: a community-based person-centred-enhanced care intervention to improve the quality of life and person-centred outcomes for people living with HIV/AIDS in Ghana—protocol for cluster randomised controlled trial
Source: BMJ Open. 2025 May 23;15(5):e102692. doi: 10.1136/bmjopen-2025-102692 (PMC12104920; doi:10.1136/bmjopen-2025-102692)
Supplement: online supplemental file 1 [file bmjopen-15-5-s001.docx]

## Appendices 1

**Consent Form for people living with HIV/AIDS for ExtraCECI cluster RCT**

**ExtraCECI: A cluster randomised controlled trial of community-based person-centred enhanced care for people living with HIV/AIDS in Ghana**

Please complete this form after you have read the Information Sheet and/ or listened to an explanation about the research.

Thank you for considering taking part in this research.

**Please tick**

The person organising the research must explain the project to you before you agree to take part. If you have any questions arising from the Information Sheet or explanation already given to you, please ask the researcher before you decide whether to join in. You will be given a copy of this Consent Form to keep and refer to at any time.

1. The research study has been explained to my satisfaction using Participant Information Sheet (V4 January 2025) and I understand what the research study involves.
2. I consent to the processing of my personal information for the purposes explained to me. I understand that such information will be treated as strictly confidential and will be handled in accordance with the terms of the Ghana Data Protection Act 2012.
3. I consent to using my contact details/information held within clinic systems and that these details can be confirmed with me at the time of consenting to be part of the study.
4. I understand that if I decide at any time during the research that I no longer wish to participate in this project, I can notify the researchers involved and withdraw from it immediately without giving any reason. Furthermore, I understand that I will be able to withdraw my data at any time up to 30^th^ April 2027.
5. I agree that the research team or other researchers may use my data for future research and understand that any such use of anonymised data would be reviewed and approved by a research ethics committee.
6. I agree to participate in this study.

**Participant’s details confirmation:**

Name------------------------------------------------------Contact----------------------------------------

*(Participant’s name and mode of contact)*

Signed -------------------------------- Date -------------------------*Researcher’s signature here*

*Indicates witness to thumbprint.*

**Researcher’s Statement:**

I ________________________________ confirm that I have carefully explained the nature, demands and any foreseeable risks (where applicable) of the proposed study to the participant.

Signed ___________________________________ Date__________________
