# Supplementary material for: ExtraCECI: a community-based person-centred-enhanced care intervention to improve the quality of life and person-centred outcomes for people living with HIV/AIDS in Ghana—protocol for cluster randomised controlled trial
Source: BMJ Open. 2025 May 23;15(5):e102692. doi: 10.1136/bmjopen-2025-102692 (PMC12104920; doi:10.1136/bmjopen-2025-102692)
Supplement: Supplementary file 2 [file bmjopen-2025-102692.reviewer_comments.pdf]

PEER REVIEW HISTORY

BMJ Open publishes all reviews undertaken for accepted manuscripts. Reviewers are asked to complete a checklist review form and are provided with free text boxes to elaborate on their assessment. These free text comments are reproduced below.

ARTICLE DETAILS

Title (Provisional)

ExtraCECI: A community-based person-centred enhanced care intervention to improve the quality of life and person-centred outcomes for people living with HIV/AIDS in Ghana: protocol for cluster randomised controlled trial.

Authors

Abboah-Offei, Mary; Elsey, Helen; Nkhoma, Kennedy Bashan; Abboah-Offei, Samson; Keding, Ada; Fenty, Justin; Yakimova, Antonina; Hewitt, Catherine; Dzansi, Gladys; Senoo-Dogbey, Vivian Efua; Ayisi Addo, Stephen; Azakili, James; Ayim, Andrews; Harding, Richard; The ExtraCECI, Project Team

---

This article was not externally reviewed at BMJ Open. Protocols that have been independently assessed prior to submission to BMJ Open are usually fast-tracked to publication on the grounds that further substantial changes will not be possible. This independent assessment will usually be external, independent review for both a substantial grant award from a non-commercial or government funder and ethics approval.
